# Supplementary material for: Assessment of health promotion behavior and associated factors among the northern Saudi adolescent population: a cross-sectional study
Source: PeerJ. 2023 Jun 27;11:e15567. doi: 10.7717/peerj.15567 (PMC10312158; doi:10.7717/peerj.15567)
Supplement: Table S1 [file peerj-11-15567-s002.docx]

Table: Exploratory Factor Analysis (EFA) – Factor loading and Factor structure of Arabic AHPS.

|  | Items | 1 | 2 | 3 | 4 | 5 | 6 |
| --- | --- | --- | --- | --- | --- | --- | --- |
| Life appreciation | Make an attempt to correct my defects | 0.640 | - | - | - | - | - |
|  | Make an effort to feel happy and content | 0.626 | - | - | - | - | - |
|  | Make an effort to know what’s important for me | 0.583 | - | - | - | - | - |
|  | Make an effort to like myself | 0.521 | - | - | - | - | - |
|  | Make an effort to feel interesting and challenged every day | 0.790 | - | - | - | - | - |
|  | Make an effort to believe that my life has purpose | 0.862 | - | - | - | - | - |
|  | I usually think positively | 0.646 | - | - | - | - | - |
|  | Make an effort to understand my strengths, weaknesses and accept them | 0.532 | - | - | - | - | - |
| Exercise | Make an effort to stand or sit up straight | - | 0.638 | - | - | - | - |
|  | Warm up before rigorous exercise | - | 0.615 | - | - | - | - |
|  | Exercise rigorously 30 minutes at least 3 times per week | - | 0.560 | - | - | - | - |
|  | Perform stretching exercise daily | - | 0.713 | - | - | - | - |
|  | Participate in physical fitness class at school weekly | - | 0.515 | - | - | - | - |
| Social support | Make an effort to have good friendships | - | - | 0.698 | - | - | - |
|  | Enjoy keeping in touch with relatives | - | - | 0.572 | - | - | - |
|  | I care about other people | - | - | 0.533 | - | - | - |
|  | Make an effort to smile or laugh every day | - | - | 0.621 | - | - | - |
|  | Talk about my troubles to others | - | - | 0.432 | - | - | - |
|  | I speak up & share my feelings with others | - | - | 0.764 | - | - | - |
|  | I talk about my concerns with others | - | - | 0.688 | - | - | - |
| Health responsibility | Read health information | - | - | 0.525 | - | - | - |
|  | Read food labels when I shop | - | - | 0.445 | - | - | - |
|  | Make an effort to choose foods without preservatives (e.g., additives on food) | - | - | 0.604 | - | - | - |
|  | Discuss my health concerns with a doctor or nurse | - | - | 0.717 | - | - | - |
|  | Observe my body at least monthly | - | - | 0.621 | - | - | - |
|  | I watch my weight | - | - | 0.703 | - | - | - |
|  | Wash hands before meals | - | - | 0.587 | - | - | - |
|  | Brush my teeth and use dental floss after meals | - | - | 0.698 | - | - | - |
| Nutrition | I eat three meals daily | - | - | - | 0.652 | - | - |
|  | Each meal includes five food groups (e.g. bread, meat, milk, fruit, vegetable) | - | - | - | 0.429 | - | - |
|  | Include dietary fiber (e.g. fruits or vegetables) | - | - | - | 0.733 | - | - |
|  | I choose foods without too much oil | - | - | - | 0.543 | - | - |
|  | Drink at least 1500 cc of water daily (or 6–8 cups) | - | - | - | 0.688 | - | - |
|  | Eat breakfast daily | - | - | - | 0.553 | - | - |
| Stress management | Make an effort to spend time daily for relaxation | - | - | - | - | 0.747 | - |
|  | Make an effort to determine the source of my stress | - | - | - | - | 0.561 | - |
|  | Make schedules and set priorities | - | - | - | - | 0.609 | - |
|  | I try not to lose control when things happen that are unfair | - | - | - | - | 0.493 | - |
|  | Make an effort to watch my mood changes | - | - | - | - | 0.677 | - |
|  | Sleep for 6–8 hours each night | - | - | - | - | 0.721 | - |
